# Special Diets and Nutrient Intakes in Morbidly Obese US Adults in Comparison to the 2020–2025 Dietary Guidelines for Americans

**DOI:** 10.1186/s12937-025-01088-7

**Published:** 2025-03-08

**Authors:** Maximilian Andreas Storz, Frieda Stübing, Roman Huber

**Affiliations:** Department of Internal Medicine II, Centre for Complementary Medicine, Medical Center, Faculty of Medicine, University of Freiburg, University of Freiburg, Freiburg, Germany

**Keywords:** Morbid Obesity, Nutrient Intake, Body Weight, Diet, Nutritional Epidemiology, Malnutrition, Sex, Macronutrients, Energy Intake

## Abstract

**Background:**

Morbid Obesity (MO), defined by a Body Mass Index (BMI) > 40 kg/m^2^, is the most severe form of obesity. The risk of suffering from any chronic medical condition is almost twice as high in MO as compared to overweight. Despite obesity being one of the most serious contemporary public health concerns, there is a paucity of nutrient intake data in adults with MO. Nutritional assessments in morbidly obese adults are often based on individuals seeking weight loss surgery rather than focusing on the general community.

**Methods:**

Using National Health and Nutrition Examination Surveys data (NHANES, 2007–2016), we estimated nutrient intakes in the general US population with MO, thereby focusing on a comparative assessment to the Dietary Guidelines for Americans (DGA, 2020–2025). Nutrient intakes were assessed in morbidly obese US adults with a BMI > 40 kg/m^2^, regardless of their intention to seek weight loss treatment and regardless of reporting special diets. Sex- and age-specific nutrient intake assessments were performed, with the aim to identify population subgroups that may warrant particular attention from a public health perspective.

**Results:**

The study sample comprised 1,708 participants with MO. This may be extrapolated to represent 14,047,276 US Americans. MO was more prevalent in females as compared to males (65.60% vs 34.40%) and the sample’s average age was 46.25 years, with a tendency towards a lower mean age in higher BMI groups. The alignment with the DGA was poor across both sexes, and particularly with regard to the nutrients of public health concern (fiber, calcium), saturated fatty acid intake and the intakes of several fat-soluble vitamins. Fiber intake was found to be particularly low in females with MO. Total energy intake was not associated with BMI in participants with MO. Morbidly obese individuals frequently reported special diets, with up to 28% of the examined population disclosing at least one special diet.

**Conclusions:**

Using a descriptive epidemiological approach, we identified numerous sociodemographic and nutritional factors associated with MO. The poor alignment with US national dietary guidelines warrants special considerations and dedicated public health nutrition efforts to combat the increasing obesity-related burden.

**Supplementary Information:**

The online version contains supplementary material available at 10.1186/s12937-025-01088-7.

## Background

The rising incidence and prevalence of obesity is a serious public health concern and exerts a significant toll on healthcare systems and societies worldwide [1, 2]. Obesity predisposes affected individuals to a wide array of adverse health conditions, and has been associated with more than 50 distinct diseases, with many of them being causally associated with an unhealthy body weight [3]. Obesity increases the risk for numerous cardiometabolic disorders, including diabetes, dyslipidemia and hypertension [3].

The rising prevalence of obesity in adolescents and young adults is of particular concern [2], as it puts these populations at risk of non-specific lower back pain and accelerated early vascular aging [4, 5]. These, in turn, both contribute to an increasing obesity-related long‐term economic burden in healthcare and to productivity losses [6]. Obesity is also a risk factor for cancer [7, 8], and was associated with an increased risk of complications during cancer treatment [9].

Morbid Obesity (MO), defined by a Body Mass Index (BMI) > 40 kg/m^2^ [10], is the most severe form of obesity, and is of particular concern from a public health nutrition perspective [11]. The risk of suffering from any chronic medical condition is almost twice as high in MO as compared to overweight [11, 12]. As the number of morbidly obese patients is continuously increasing, and since more and more individuals present for weight loss treatment, there is a greater need to understand their dietary habits [13]. It is well known that morbidly obese individuals who undergo bariatric surgery often present with nutritional deficiencies [14–16]. Ciobârcă et al. reviewed micronutrient deficiencies in severely obese individuals, and found high deficiency rates for vitamin B12, iron and folate [17]. Notably, not every individual with MO presents for bariatric surgery, and the reviewed nutritional data in the literature often stems from specialized institutions (e.g., weight-loss surgery clinics) which may introduce selection and reporting bias.

In this context, Harbury et al. called for additional high-quality nutrition studies dedicated to collecting comprehensive dietary data from individuals with MO [13]. Harbury et al. also emphasized the need to extend nutritional assessments to individuals from the general community rather than exclusively focusing on individuals seeking weight loss surgery. The authors also called for a consistent reporting of nutrient intake data by sex to enhance the general understanding of nutritional deficiencies in MO.

While researchers began to address this call [18], few studies focused exclusively on morbidly obese individuals. Using data from the National Health and Nutrition Examination Surveys (NHANES), we estimated nutrient intakes in the general US population with MO, thereby focusing on a comparative assessment to established national dietary guidelines, such as the Dietary Guidelines for Americans (DGA) [19]. The aim of this study was to estimate nutrient intakes in morbidly obese individuals with a BMI > 40 kg/m^2^, regardless of their intention to seek weight loss treatment or not. Sex- and age-specific nutrient intake assessments were performed, with the goal to identify population subgroups that may warrant particular attention from a public health nutrition perspective.

## Methods

### Data source and study type

This work was performed with data from the NHANES – a nationally representative survey of the US civilian, noninstitutionalized population [20–22]. The overarching goal of this program is the assessment of the health and nutritional status of adults and children in the US through a combination of in-home personal interviews, physical health examinations, and laboratory tests in specialized mobile medical examination centers [20]. The NHANES interview includes a large set of standardized questions regarding sociodemographic data, diet and health behavior as well as acute and chronic health conditions [21, 22]. The examination component includes an anthropometric assessment as well as a dental examination and vision testing. The survey’s hallmark is the special (complex, multistage, clustered) sampling design that uses unequal probabilities of selection. Thus, special analysis techniques are required (see statistical analysis section below) [21]. The NHANES permits its user to compute nationally representative statistics. Said estimation procedures include inflation by the reciprocal of the probability of selection, adjustment for nonresponse, and post stratified ratio adjustment to population totals [21]. Five consecutive NHANES cycles were appended for this analysis (2007/2008–2015/2016) to increase the unweighted sample size.

### Methodological approach and primary outcome

The methodology for this descriptive nutritional epidemiology study type has been discussed previously [23–25]. In brief, it follows the following three step pattern: I) a nutrient intake estimation in NHANES participants with MO (defined by a BMI > 40 kg/m^2^); II) a comparison to established national dietary guidelines (the current edition of the DGA [19]); III) age- and sex-specific analyses to identify population strata of special concern. The primary outcome was nutrient intake, and included 26 nutrients as specified in the DGA Table A2-1 covering Daily Nutrition Goals (DNG) [19]. The secondary outcome included a descriptive comparison to the DGA (binary descriptive analysis, specific nutrient goal met on average: yes/no).

The most recent edition of the DGA was released in a joint effort by the US Departments of Health and Human Services (DHHS) and Agriculture (USDA) [26]. As discussed by DeSalvo et al., the DGA are an important tool of a complex and multifaceted national strategy to promoting health and to preventing diet-related chronic diseases, such as obesity, in the US [26]. As national guidelines, the DGA are considered an evidence-based foundation for federal government nutrition education materials [26]. While the DGA serve as a larger framework that could be customized to individual needs, they generally emphasize nutrient-dense foods to ensure an adequate intake of critical vitamins, minerals, and other health-promoting nutrients [27]. A special focus of the current DGA edition is the life span approach, with specific dietary recommendations for every stage of life from infancy through older adulthood [28]. As such, the DNG are displayed for three age groups in adults: adults aged 19–30 years; adults aged 31–50 years and adults aged 51 + years. We employed a similar classification and descriptively compared nutrient intakes in morbid obsese adults with the DNG stratified by age–sex groups. The DNG in the current DGA stem from different sources, which have been described earlier in great detail [23, 24].

### Anthropometric data

Anthropometric data was obtained from the NHANES examination data section (body measures module). The NHANES body measures data was collected in the mobile examination center by trained health technicians [29]. Weight and height were measured; self-reported data was not accepted. Only participants with MO were considered (defined by a BMI > 40 kg/m^2^) for this article. In a first step, nutrient intakes were computed for the whole sample. Subsequently, we constructed four BMI subgroups as follows: I) BMI: 40–44.99 kg/m^2^, II) BMI: 45–49.99 kg/m^2^, III) BMI: 50–54.99 kg/m^2^, and IV) BMI: > 55 kg/m^2^. Nutrient intakes were then estimated and subsequently compared across the 4 groups.

### Nutrient intake data and special diets

Nutrient intake data was obtained from the NHANES dietary interview [30]. The underlying methods have been described earlier in detail [31–33]. We exclusively focused on nutrients from foods and beverages. Nutrients obtained through dietary supplements were not considered. The nutrient intake assessment was conducted in person by trained dietary interviewers fluent in Spanish and English. It was based on a computerized 24 h single dietary recall method to estimate energy and nutrient intakes for all participants. Following a previous approach, we also registered special diets to investigate the weighted percentage of participants with MO who reported a special diet [34]. Only individuals with a reliable dietary status who met the NHANES minimum criteria were considered [30]. However, for an unbiased, non-selective and generally valid approach, we did *not* specify total energy intake criteria for this analysis. Unlike in previous investigations [25], we also considered individuals with an energy intake below 800 kcal/d or above 5000 kcal/d. Energy intake is of particular importance in obese individuals and was thus a main point of interest for this study [35]. The rationale was to consider both individuals on a special diet (e.g. on an energy-restricted weight loss diet) and participants with a very high energy intake (considering that we investigated participants with MO). Scatterplots were constructed to visualize energy intakes of participants.

### Covariat﻿es

Important covariates included sex (categorical variable, two categories: male / female), age (continuous variable), income (categorical variable, two categories: < 20,000 US$ / ≥ 20,000 US$), marital status (categorical variable, three categories: living with partner or married / divorced or separated or widowed / never married), ethnicity/race (categorical variable, five categories: Mexican American / Other Hispanic / Non-Hispanic White / Non-Hispanic Black / Other Race), highest educational achievement (categorical variable, five categories: less than 9th grade / 9-11th grade / high school graduate / some college or AA degree / college graduate or above), alcohol intake status (categorical variable, two categories: ≥ 12 drinks per year: yes / no), and smoking status (categorical variable, three categories: non-smoker / current smoker / former smoker).

## Statistical analysis

The statistical analysis was performed with STATA 14 statistical software (StataCorp. 2015. Stata Statistical Software: Release 14. College Station, TX: StataCorp LP). Survey commands (svyset and svy) were used to account for the special NHANES survey design. For this analysis, we constructed an appropriate 10-year survey weight (2007–2016) for dietary data to obtain reliable weighted percentages adjusted to the US adult population.

The statistical analysis followed previously established procedures [23, 24, 31]. Normally distributed (continuous) variables were shown with their mean and corresponding 95%-CI (confidence interval). For categorical variables, we reported weighted proportions with their corresponding 95%-CI in parenthesis. As done earlier [31, 36], the reliability of all estimated proportions was checked as recommended in the 2017 NCHS data presentation guidelines [37]. Unreliable proportions/estimations were clearly flagged. For the comparison between BMI groups, we used regression analyses followed by adjusted Wald tests. Statistical significance was determined at α = 0.05. The nutrient intake comparison to the DGA was performed in a descriptive manner. Associations between BMI category and categorical sociodemographic variables were assessed with Stata’s design-adjusted Rao-Scott test.

Finally, we constructed multivariate regression models to predict participants’ total energy intake after adjustment for important sociodemographic covariates. Post regression, we used Stata’s marginsplots function to visualize statistics from fitted models and to display marginal predicted values.

## Results

After excluding participants with missing data (see Supplementary Fig. 1), the final sample comprised *n* = 1,708 participants with MO. This may be extrapolated to represent *n* = 14,047,276 Americans with MO. Table 1 displays the sample’s characteristics. The majority of participants reported a BMI between 40 and 44,99 kg/m^2^, however, the sample also comprised *n* = 303 unweighted observations with a BMI ≥ 50,00 kg/m^2^.

**Table 1:** Sample characteristics by BMI category

	Whole Sample *(n* = *1,708)*	BMI: 40–44.99 *(n* = *982)*	BMI: 45–49.99 *(n* = *423)*	BMI: 50–54.99 *(n* = *169)*	BMI: > 55 *(n* = *134)*	***p-value***
**Sex**						*p* = 0.307^b^
Male	34.40% (31.68–37.22)	36.68% (32.54–41.03)	31.74% (25.86–38.26)	32.60% (25.53–40.56)	28.22% (19.59–38.82)	
Female	65.60% (62.78–68.32)	63.32% (58.97–67.46)	68.26% (61.74–74.14)	67.40% (59.44–74.47)	71.78% (61.18–80.41)	
**Age (years)**	46.25 (45.19–47.31)	47.38 (46.17–48.58)	44.85 (42.96–46.73)	45.27 (41.59–48.96)	43.65 (40.27–47.02)	***p* = 0.043**^c^
**BMI (kg/m**^**2**^**)**	45.71 (45.36–46.05)	42.11 (41.97–42.24)	47.17 (47.00–47.33)	51.97 (51.50–52.43)	60.81 (59.84–61.78)	***p* < 0.001**^c^
**Marital status**						*p* = 0.763^b^
Living with partner/married	58.89% (54.94–62.73)	60.12% (56.30–63.83)	56.49% (49.31–63.41)	56.85% (46.05–67.04)	60.39% (47.56–71.93)	
Divorced/separated/widowed/	20.67% (18.25–23.32)	20.87% (17.85–24.25)	21.90% (16.59–28.32)	18.57% (13.09–25.68)	17.50% (10.40–27.94)	
Never married	20.43% (17.55–23.65)	19.01% (16.04–22.39)	21.61% (16.38–27.96)	24.57% (16.57–34.83)	22.11% (14.94–31.45)	
**Annual household income**						***p* = 0.019**^b^
< 20,000 US$	18.78% (15.82–22.15)	16.02% (12.71–20.01)	21.17% (16.52–26.72)	20.96% (13.73–30.64)	29.21% (20.26–40.13)	
≥ 20,000 US$	81.22% (77.85–84.18)	83.98% (79.99–87.29)	78.83% (73.28–83.48)	79.04% (69.36–86.27)	70.79% (59.87–79.74)	
**Ethnicity/race**						*p* = 0.629^b^
Mexican American	10.06% (7.35–13.61)	9.80% (6.97–13.61)	10.65% (6.51–16.97)	9.92% (5.28–17.89)✝	10.12% (4.73–20.33)✝	
Other Hispanic	5.17% (3.91–6.81)	5.07% (3.60–7.11)	5.12% (3.01–8.56)	5.59% (2.63–11.49)✝	5.54% (2.69–11.07)✝	
Non-Hispanic White	60.86% (55.43–66.03)	63.28% (57.01–69.12)	56.90% (48.56–64.86)	60.75% (49.33–71.10)	56.00% (45.43–66.06)	
Non-Hispanic Black	20.07% (16.52–24.16)	18.29% (14.64–22.61)	22.36% (17.15–28.60)	22.74% (15.57–31.95)	22.57% (16.08–30.71)	
Other Race ^a^	3.84% (2.65–5.54)	3.56% (2.26–5.57)	4.97% (2.73–8.90)	0.99% (0.23–4.22)	5.78% (2.59–12.38)✝*	
**Educational level**						***p* = 0.030**^b^
Less than 9th grade	4.33% (3.36–5.55)	4.85% (3.50–6.69)	4.53% (2.74–7.41)	2.95% (1.60–5.38)✝	1.23% (0.27–5.33)*	
9-11th grade	12.28% (10.79–13.95)	10.83% (8.76–13.32)	12.47% (9.54–16.12)	16.64% (9.93–26.56)	17.48% (10.83–26.98)	
High school graduate/GED ^d^	23.92% (21.39–26.65)	22.76% (19.49–26.38)	22.48% (17.46–28.45)	32.17% (20.68–46.31)	27.60% (19.21–37.92)	
Some college or AA degree ^e^	40.57% (37.22–44.01)	39.98% (36.05–44.05)	40.89% (33.89–48.28)	38.61% (26.93–51.76)	46.63% (35.91–57.67)	
College graduate or above	18.89% (15.98–22.20)	21.58% (17.93–25.74)	19.63% (14.41–26.17)	9.63% (5.60–16.06)	7.07% (3.81–12.73)✝*	
**Self-reported special diet**						*p* = 0.519^b^
Yes	27.67% (24.78–30.77)	26.03% (22.72–29.63)	30.08% (24.19–36.70)	30.73% (22.37–40.57)	28.18% (20.13–37.93)	
No	72.33% (69.23–75.22)	73.97% (70.37–77.28)	69.92% (63.30–75.81)	69.27% (59.43–77.63)	71.82% (62.07–79.87)	
**12 alcoholic drinks per year**						*p* = 0.559^b^
No	31.61% (27.77–35.72)	32.24% (27.29–37.62)	30.49% (23.84–38.08)	26.75% (19.03–36.20)	36.86% (26.67–48.40)	
Yes	68.39% (64.28–72.23)	67.76% (62.38–72.71)	69.51% (61.92–76.16)	73.25% (63.80–80.97)	63.14% (51.60–73.34)	
**Smoking status**						*p* = 0.183^b^
Non-smoker	57.98% (54.57–61.31)	56.05% (51.94–60.08)	62.43% (55.09–69.24)	60.09% (47.03–71.85)	54.62% (43.65–65.16)	
Current smoker	16.91% (14.53–19.60)	16.95% (14.14–20.20)	17.89% (13.50–23.33)	18.59% (11.79–28.05)	11.00% (6.23–18.71)	
Former smoker	25.11% (22.71–27.66)	27.00% (23.36–30.97)	19.67% (14.46–26.19)	21.33% (13.41–32.18)	34.37% (23.78–46.79)	

Table 1 Summarizes sample characteristics. All shown proportions are weighted proportions with their 95%-CI in parenthesis. Continuous variables (age and BMI) are shown as means with their 95%-CI. The total number of underlying unweighted observations was *n* = 1,708. The “✝” symbol indicates an unreliable proportion, as per the recent National Center for Health Statistics Guidelines [37]. * indicates significant between group differences in the estimated weighted proportions.

**a** = includes multi-racial; **b** = *p*-value based on Stata’s design-adjusted Rao–Scott test; **c** = *p*-value based on regression analyses followed by adjusted Wald tests; **d** = GED = General Educational Development diploma; **e** = Associate of Arts degree

MO was more prevalent in females as compared to males (65.60% vs 34.40%, weighted proportions). The sample’s average age was 46.25 years, with a tendency towards a lower mean age in higher BMI groups. The proportion of individuals with an annual household income < 20,000 US$ increased in the higher BMI groups. Conversely, the proportion of college graduate participants decreased in the higher BMI groups. A reservation must be made, that not all weighted proportions could be considered reliable as per the recent NCHS data presentation standards [37]. Unreliable proportions were clearly flagged.

Special diets were popular among participants with MO, with approximately 28% of participants reporting adherence to at least some kind of special diet. The unweighted number of participants reporting a particular special diet is shown in Supplementary Table 1. Weight loss and low calorie diets were the most popular reported special diets in the examined sample (*n* = 288 unweighted observations). Reporting of a special diet was not independent of sex (*p* = 0.020), with 30.46% of morbidly obese females reporting a special diet as opposed to 22.37% males (*p* = 0.012 for the difference in the weighted proportion).

Table 2 displays nutrient intake data by BMI category. With the exception of vitamin C, no significant between group differences were observed. Mean energy intake ranged from 2082.90 kcal/day in those with a BMI of 40–44.99 to 2188.67 kcal/d in those with a BMI of 50–55.99. Nutrient intake data for the entire sample is shown in Supplementary Table 2, whereas Supplementary Table 3 shows data by sex.

**Table 2:** Nutrient intake data by BMI category

BMI Category (n)	BMI: 40–44.99 *(n* = *982)*		BMI: 45–49.99 *(n* = *423)*		BMI: 50–54.99 *(n* = *169)*		BMI: > 55 *(n* = *134)*		
	mean	CI	mean	CI	mean	CI	mean	CI	*p*
Energy intake (kcal/d)	2082.90	2001.65–2164.15	2152.24	2037.63–2266.86	2188.67	2016.88–2360.47	2115.55	1946.76–2284.34	0.567
Protein (%kcal)	16.18	15.68–16.68	15.43	14.79–16.08	16.81	16.03–17.59	16.08	14.84–17.31	0.053
Protein (g)	81.38	77.69–85.08	81.16	76.66–85.66	87.55	79.69–95.42	82.61	75.46–89.75	0.503
Carbohydrate (%kcal)	47.94	46.90–48.93	49.12	47.67–50.56	47.69	45.35–50.03	45.99	43.98–48.00	0.127
Carbohydrate (g)	246.76	236.76–256.76	260.81	246.97–274.65	262.74	239.92–285.56	242.26	222.76–261.77	0.102
Fiber (g)	15.98	14.93–17.03	15.82	14.44–17.20	16.18	14.21–18.15	14.63	12.91–16.34	0.488
Total lipid (%kcal)	35.10	34.03–36.16	35.13	33.95–36.32	34.86	32.38–37.35	37.68	35.92–39.44	0.099
Saturated Fatty Acids (%kcal)	11.56	11.12–12.00	11.57	11.05–12.08	11.04	10.14–11.94	12.32	11.54–13.11	0.167
18:2 Linoleic acid (g)	16.51	15.32–17.69	17.40	15.82–18.98	17.12	15.22–19.02	18.23	15.79–20.68	0.617
18:3 Linolenic acid (g)	1.69	1.55–1.83	1.81	1.64–1.97	1.75	1.54–1.97	1.80	1.58–2.02	0.747
Calcium (mg)	921.57	876.10–967.04	964.45	890.10–1038.80	907.05	799.59–1014.50	958.09	833.43–1082.75	0.757
Iron (mg)	14.16	13.40–14.92	13.79	12.84–14.74	16.30	14.30–18.30	14.50	12.92–16.08	0.111
Magnesium (mg)	279.82	264.88–294.76	277.24	259.14–295.35	284.31	256.61–312.00	263.36	242.54–284.19	0.556
Phosphorus (mg)	1339.64	1278.14–1401.14	1349.76	1268.47–1431.04	1385.58	1264.71–1506.45	1325.48	1195.89–1455.08	0.889
Potassium (mg)	2551.22	2409.99–2692.45	2506.71	2349.71–2663.71	2590.06	2294.12–2886.00	2362.04	2131.28–2592.79	0.600
Sodium (mg)	3539.23	3383.99–3694.47	3669.96	3402.31–3937.60	3807.38	3436.62–4178.13	3753.45	3426.94–4079.96	0.374
Zinc (mg)	10.94	10.38–11.50	10.89	10.15–11.64	11.53	9.81–13.25	11.47	10.34–12.60	0.726
Vitamin A (RAE mcg)	585.96	541.11–630.80	592.67	528.58–656.76	584.20	456.52–711.88	525.34	443.90–606.79	0.496
Vitamin E (mg AT)	7.89	7.30–8.48	8.00	7.24–8.76	8.85	7.76–9.94	7.37	6.44–8.31	0.169
Vitamin D (IU)	159.62	139.53–179.71	170.00	144.71–195.29	158.09	130.11–186.06	156.07	116.68–195.46	0.886
Vitamin C (mg)	68.93	61.29–76.56	85.82	74.47–97.16	71.69	54.30–89.08	59.91	42.94–76.89	**0.023**
Thiamin (mg)	1.52	1.45–1.59	1.53	1.42–1.63	1.60	1.43–1.77	1.62	1.40–1.85	0.669
Riboflavin (mg)	2.07	1.98–2.17	2.00	1.85–2.14	2.11	1.85–2.36	1.96	1.72–2.19	0.644
Niacin (mg)	24.70	23.54–25.87	24.12	22.65–25.59	27.99	23.99–31.99	24.79	22.41–27.17	0.316
Vitamin B-6 (mg)	1.94	1.82–2.06	1.83	1.68–1.97	2.18	1.76–2.60	1.76	1.58–1.94	0.172
Vitamin B-12 (mcg)	5.06	4.61–5.51	4.56	4.10–5.01	4.98	4.16–5.80	4.89	4.18–5.60	0.356
Choline (mg)	326.40	307.55–345.24	318.13	294.82–341.45	335.31	298.98–371.64	304.21	271.36–337.06	0.591
Vitamin K (mcg)	107.72	92.63–122.81	110.85	97.94–123.77	94.29	75.93–112.65	106.15	67.33–144.97	0.494
Folate (mcg DFE)	500.40	468.57–532.22	492.79	452.65–532.92	548.78	462.70–634.86	453.53	399.54–507.53	0.315

Table 2 Summarizes nutrient intake data by BMI category and is based *n* = 1,708 unweighted observations. A *p*-values < 0.05 indicates significant intake difference between the 4 examined BMI groups. All *p*-values in Table 2 are based on regression analyses followed by adjusted Wald tests

Table 3 displays macronutrient and fiber intake in morbidly obese males compared to the DNG in the 2020–2025 DGA stratified by age group. In a similar style, Table 4 shows macronutrient and fiber intake in morbidly obese females compared to the DNG in the 2020–2025 DGA stratified by age group. As for the examined males, a poor alignment with the DGA was observed. All examined age groups exceeded the recommended maximum daily saturated fat intake whereas none of the groups met the recommendations for fiber. A comparable pattern was observed in females.

**Table 3:**
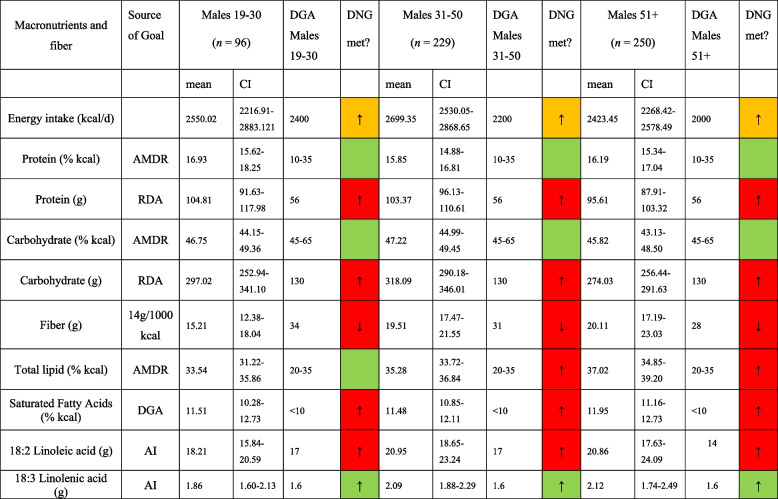
Macronutrient and fiber intake in morbidly obese males in comparison to the daily nutritional goals in the 2020–2025 DGA, stratified by age group

*AMDR* Acceptable Macronutrient Distribution Range, *RDA* Recommended Dietary Allowance, *AI* Adequate Intake; based on [19]. *DNG* Daily Nutritional Goals. Table 3 is based on *n* = 575 unweighted observations

**Table 4:**
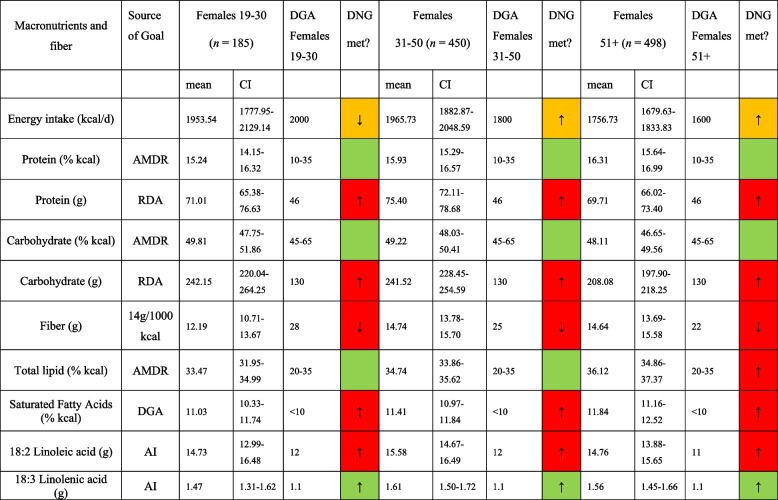
Macronutrient and fiber intake in morbidly obese females in comparison to the daily nutritional goals in the 2020–2025 DGA. stratified by age group

*AMDR* Acceptable Macronutrient Distribution Range, *RDA* Recommended Dietary Allowance, *AI* Adequate Intake; based on [19]. *DNG* Daily Nutritional Goals. Table 4 is based on *n* = 1133 unweighted observations

Mineral and vitamin intakes in morbidly obese males and females are shown in Tables 5 and 6, respectively. Again, the DNG was not met for many nutrients in both sexes, including magnesium, potassium, vitamins A, D and E, and vitamin C (except for males aged 31–50 years). Both sexes reported a daily sodium intake far above the chronic disease risk reduction intake recommendation of 2300 mg per day. While the daily intake of water-soluble vitamins appeared to be adequate in both sexes, an insufficient vitamin K intake was observed in males and females aged 19–30 years.

**Table 5:**
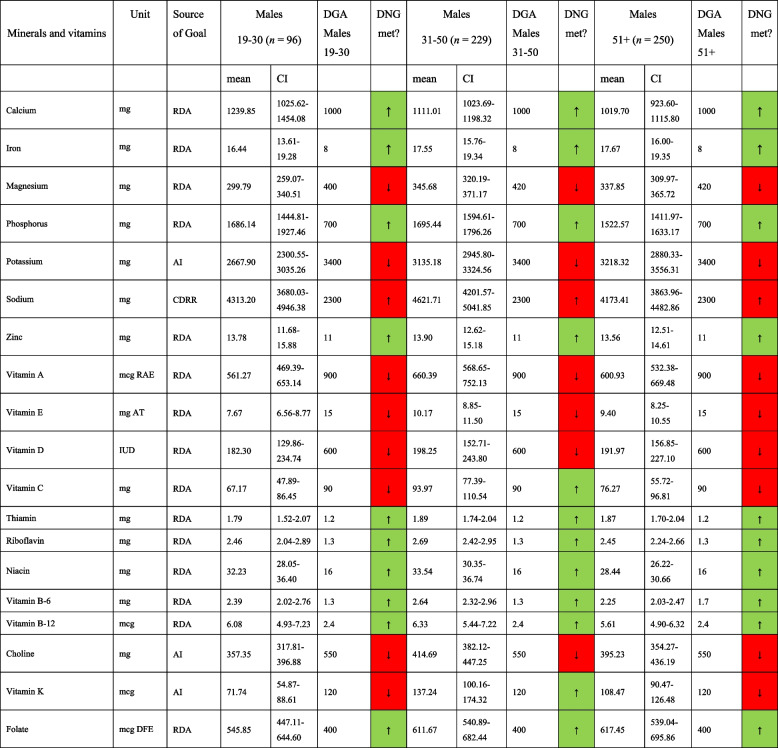
Mineral and vitamin intake in morbidly obese males in comparison to the daily nutritional goals in the 2020–2025 DGA, stratified by age group

*AMDR* Acceptable Macronutrient Distribution Range, *RDA* Recommended Dietary Allowance, *CDRR* Chronic disease risk reduction intake, *AI* Adequate Intake; based on [19]. *DNG* Daily Nutritional Goals. Table 5 is based on *n* = 575 unweighted observations

**Table 6:**
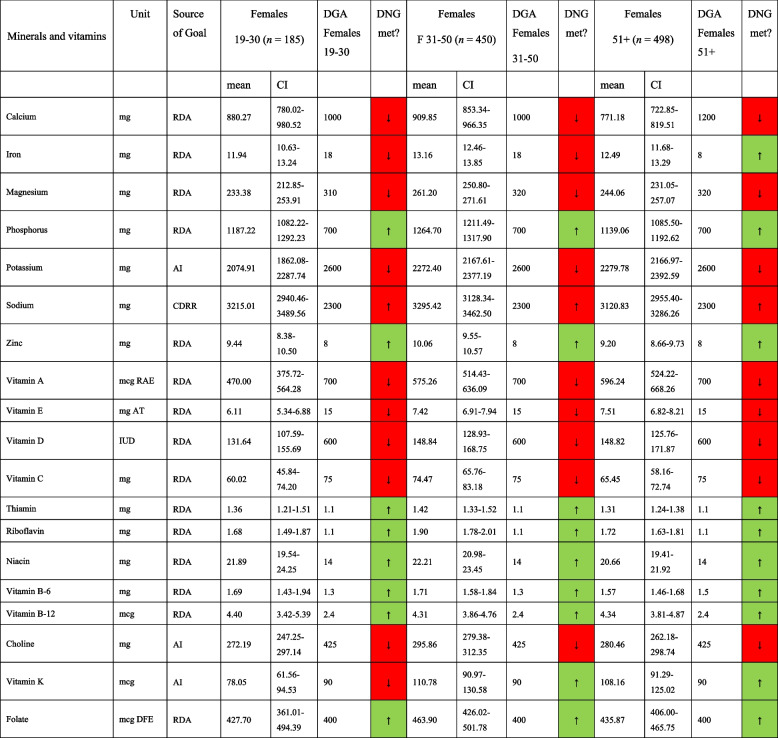
Mineral and vitamin intake in morbidly obese females in comparison to the daily nutritional goals in the 2020–2025 DGA, stratified by age group

*AMDR* Acceptable Macronutrient Distribution Range, *RDA* Recommended Dietary Allowance, *CDRR* Chronic disease risk reduction intake, *AI* Adequate Intake; based on [19]. Table 6 is based on *n* = 1133 unweighted observations

In a subsequent step, we compared nutrient intakes between those who reported a special diet and those who denied a special diet (see Supplementary Table 4). When looking at the whole sample, numerous significant between group differences were observed, e.g., for the total energy intake, for protein intake, carbohydrate intake, linoleic acid intake, phosphorus intake, thiamin intake and niacin intake. No consistent conclusions could be drawn with regard to the overall alignment with the DGA when comparing participants with a special diet to those denying a special diet. For example, despite a lower total energy intake, participants on a special diet reported higher non-energy adjusted intakes of fiber. Then again, these participants also reported a higher total fat intake (in percent of total energy intake). Despite significant between group-differences, both groups were characterized by an overall poor DGA alignment and the alignment varied from nutrient to nutrient.

A sex-specific comparison (shown in Supplementary Tables 5 and 6) suggested that special diets may at least partially alter nutrient intakes in a sex-specific manner. Special diets resulted in a significantly lower intake of carbohydrates and calcium in males. The lower carbohydrate intake in females on a special diet was accompanied by a significantly lower sodium intake. Energy intake was lower in individuals of both sexes who reported a special diet.

**Fig. 1:**
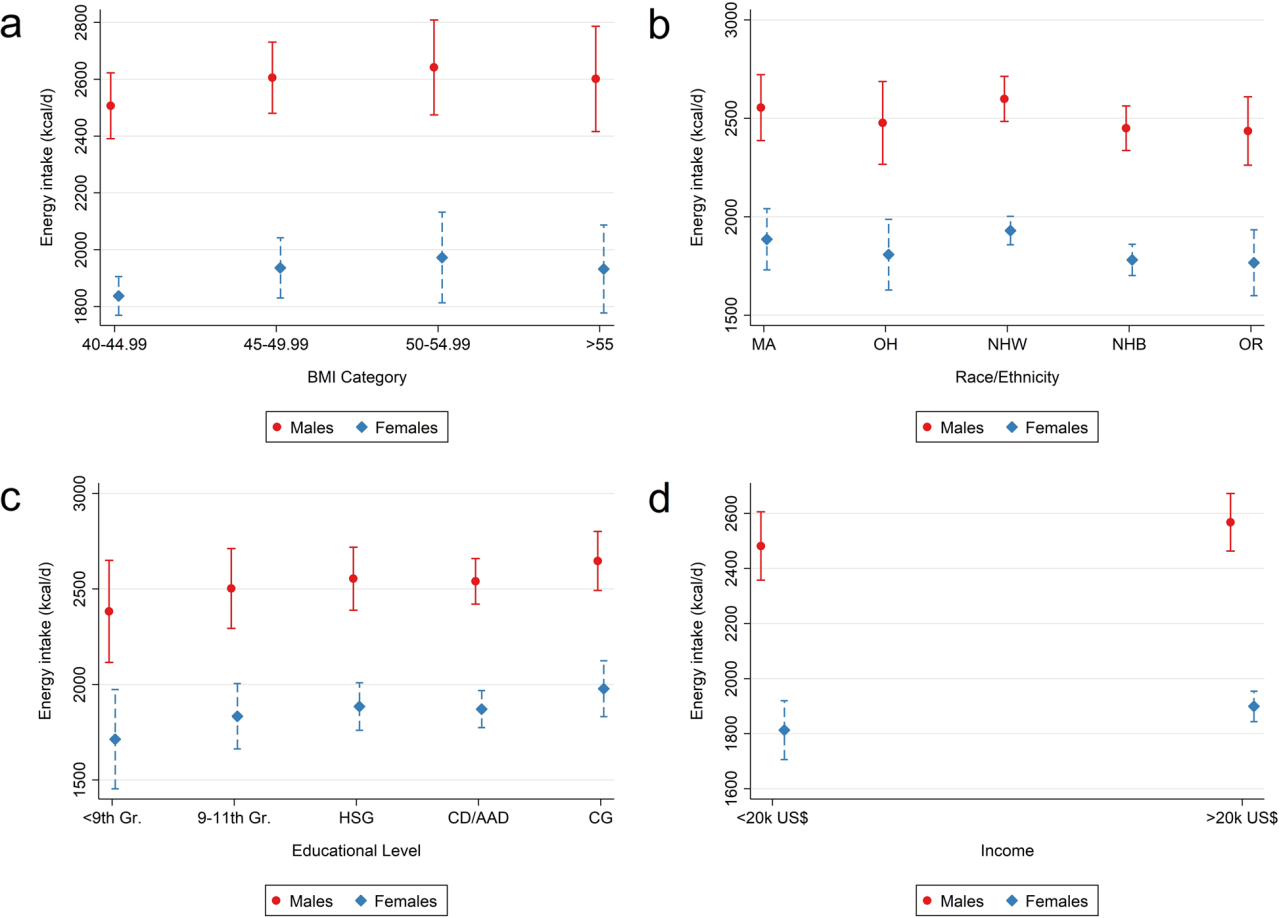
Marginsplots: Predictive margins for the total daily energy intake (in kcal). Figure 1 legend: **a** = Plot of marginal predicted values for the daily energy intake based on a multivariable regression model adjusting for race/ethnicity (categorical), age (continuous), BMI (categorical), sex (categorical), educational level (categorical) and income (categorical). **b** = plot of marginal predicted values for the daily energy intake, illustrating differences in the relationship of energy intake and sex (male/female) depending on race/ethnicity. **c** = plot of marginal predicted values for the daily energy intake, illustrating differences in the relationship of energy intake and sex (male/female) depending on the educational level. **d** = plot of marginal predicted values for the daily energy intake, illustrating differences in the relationship of energy intake and sex (male/female) depending on income. MA = Mexican American; OH = Other Hispanic; NHW = Non-Hispanic White; NHB = Non-Hispanic Black; OR = Other Race. Gr = Grade; HSG = High School Graduate; CD = College Degree; AAD = Associate Degree; CG = College Graduate

We then constructed multivariate linear regression models to examine potential associations between energy intake and BMI category. All models adjusted for race/ethnicity (categorical), age (continuous), BMI (categorical), sex (categorical), educational level (categorical) and income (categorical). Marginal predicted values were graphed from these models. Figure 1 (panel a) displays marginal predicted values for daily energy intakes depending on sex for each BMI category. In a similar style, marginal predicted energy intake values are shown by race/ethnicity (panel b), educational level (panel c) and income (panel d). Significant differences between ethnicities were found for non-Hispanic Whites and non-Hispanic Blacks (-149.07 kcal/d [-254.68—(-43.45); p-value: 0.006] after adjustment for covariates. The difference remained significant after adjustment for multiple comparisons (p-value after Bonferroni-correction: 0.025; contrasts after adjustments: -149.07 kcal/d [-284.70—(-13.43)]. No significant differences were found between the different educational levels. The underlying statistical model is displayed in Supplementary Table 7.

**Fig. 2:**
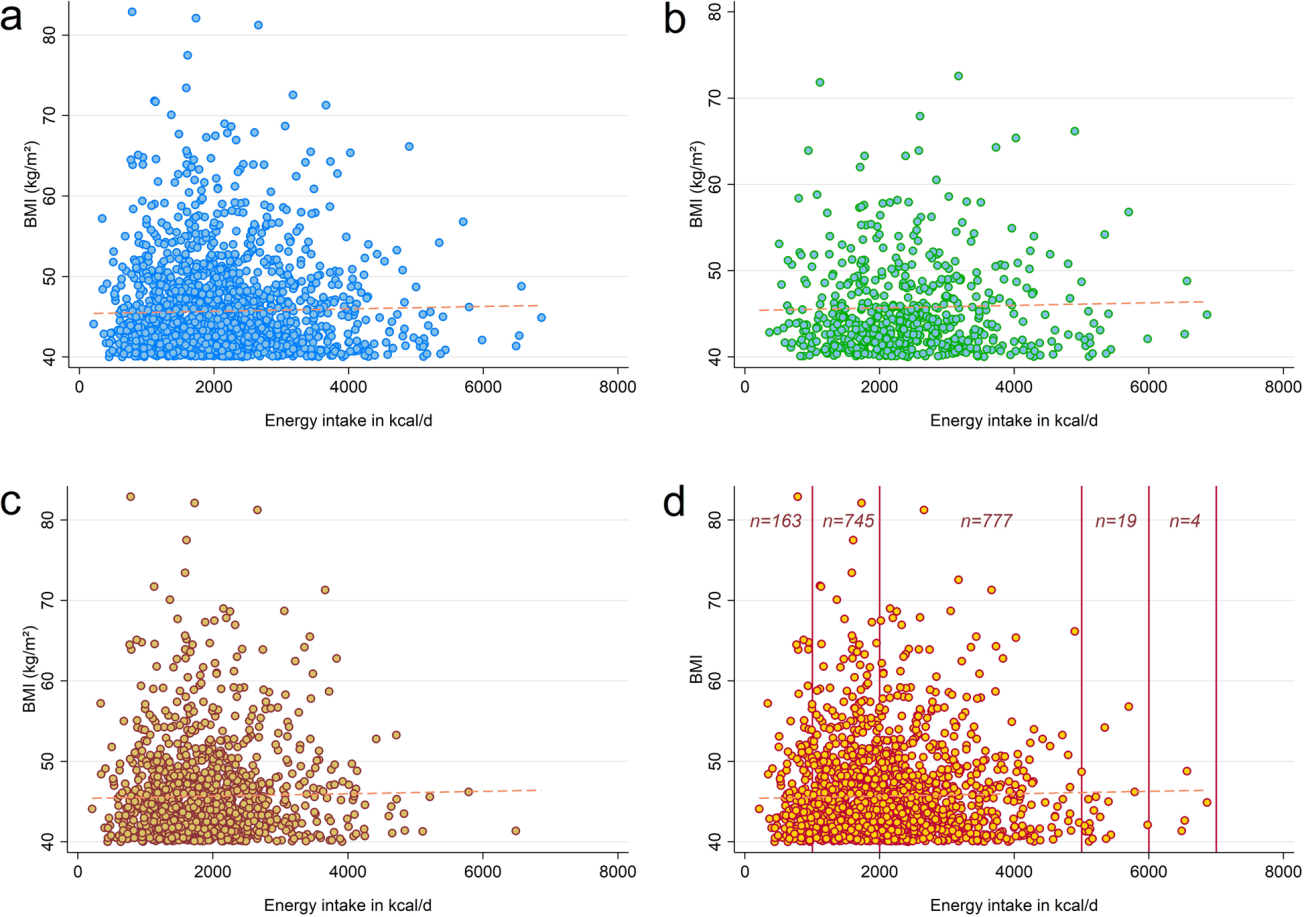
Scatter plots displaying the relationship between energy intake (in kcal/d) and BMI (in kg/m^2^). Figure 2 legend: **a** = entire sample; **b** = males only (*n* = 575); **c** = females only (*n* = 1133); **d** = entire sample with energy intake categories

Ultimately, we constructed scatterplots to visualize the relationship between energy intake and BMI. Figure 2, panel a, displays this relationship in the entire sample. Panels b and c are restricted to men and women, respectively. Panel d shows the unweighted number of observations in five pre-defined energy intake ranges. Only *n* = 23 participants reported an energy intake above 5000 kcal/d, whereas *n* = 163 participants reported energy intakes below 1000 kcal/d.

## Discussion

The present study investigated nutrient intake data in NHANES participants with MO with a special emphasis on the alignment with the current DGA. Five NHANES cycles were analyzed including data from *n* = 1,708 participants with MO (which may be extrapolated to represent *n* = 14,047,276 Americans). Several key findings emerged which may be summarized as follows:

I) The prevalence of MO was substantially higher in females as compared to males. II) The alignment with the DGA was poor across both sexes, and particularly with regard to saturated fatty acid intake, fiber intake and the intake of several fat-soluble vitamins. III) Energy intake was not associated with BMI in NHANES participants with MO and varied considerably within the sample, with many participants reporting a daily energy intake below 1000 kcal. IV) NHANES participants with MO frequently reported special diets, with up to 28% of the examined population reporting at least one special diet. V) Nutrient intakes in individuals on a special diet differed significantly from those denying a special diet, potentially mediated by the lower total energy intake.

Considering the special approach for this analysis, a careful discussion in the context of the available literature is warranted. Our data is in line with previous NHANES data, showing that MO is much more prevalent in females than in males [38]. There has been a growing awareness about the importance of understanding sex differences in obesity [39]. Obesity-related sex disparities may result from differences in the socioeconomic status in males and females as well as from a mismatch in sociocultural factors [40]. Feeding practices in the postpartum period, hormone-related comorbidities and divergent customs may also a play a pivotal role [40]. While it is beyond the scope of this descriptive epidemiological analysis to analyze and control for these factors, it must be kept in mind that women comprised almost 66% of the weighted sample [40]. Such information is crucial for public health nutrition strategies involving participants with MO.

The alignment with the DGA was poor across all sexes and ages group. This applied in particular to the so-called nutrients of public health concern, including fiber, calcium, and potassium. None one of the examined age- and sex-subgroups had a mean intake that was in line with the DGA. Dietary fiber plays a pivotal role in the prevention of obesity and obesity-related chronic diseases [41]. As summarized by Waddell and Orfila, numerous direct and indirect mechanisms linking inadequate fiber intakes and obesity have been identified, and a low fiber intake may be causally involved in the development of obesity [41]. Mechanisms include (but are not limited to) altered digestion and nutrient absorption, the stimulation of gut hormones such as glucagon-like-peptide-1, and a reduced appetite [41]. It is well known that nearly 19 out of 20 Americans do not consume the minimum recommended amount of daily fiber [42], and further efforts are warranted to increase fiber intake in the United States [31]. Our data reiterate this urgent need and confirm that fiber intake is of particular concern in Americans with MO. Likewise, the low intakes of dietary magnesium and potassium are of similar concern, as both were associated with a lower body fat content [43]. 

A reservation must be made, that we aimed for an analysis approach that minimized potential selection bias. Considering the fact that we included participants with special diets (e.g. energy-deficient weight loss diets), we also had *n* = 163 unweighted observations with an energy intake < 1000 kcal/d. This may have partly contributed to the abovementioned deficient intakes of several nutrients. Then again, our data describes the unbiased *status quo* of nutrient intakes. From a nutritional epidemiology perspective, however, energy intakes below < 800 kcal/d may indicate underreporting or recall bias [44]. One could go as far as arguing that such energy intakes are implausible in morbidly obese adults, and cannot be sustained over a longer period.

It is widely accepted that self-reported dietary intake is generally lower than habitual dietary intake and underreporting occurs more often in heavier individuals compared to leaner ones [45, 46]. Against this background, it is plausible that the herein presented data was also subject to underreporting and several other biases. Marginal predicted energy intakes in our study, however, were in line with findings from a systematic review investigating dietary intakes in obese individuals undergoing either sleeve gastrectomy or gastric bypass surgery [47]. The weighted mean of total energy intake *prior* to surgery in said systematic review was 2049 kcal/day [47]. Above all, it remains surprising that energy intake was not positively associated with BMI in our study. A study involving 14,281 Greece adults from the early 2000s reported that an increment of about 500 kcal intake was found to correspond to an increment of about 0.33 kg/m^2^ of BMI (after adjustments for age and gender). Notably, this finding applied to non-dieting individuals only, and energy under-reporters were excluded from that particular analysis [48]. We, on the other hand, included individuals with special diets (e.g., weight-loss diets) to reflect the actual nutrient intake data in this cohort as they exist. Said approach could potentially explain the lack of an association between energy intake in BMI in our study. Another explanation could be an underlying underreporting by participants with a higher BMI.

One could also criticize our approach by suggesting that the inclusion of special diets and potential under-reporters introduces bias on its own. In fact, this could potentially have led to an underestimation of the total long-term nutrient intake. Then again, it is well known that underreporting of food intake seems to be more of a concern for *specific* food items, which are generally considered to be “bad for health”, such as foods high in processed fats [45]. This may not be the case for potassium and fiber-rich plant-foods, which were most likely under-consumed in the examined sample. As such, the approach may be adequate and the findings legitimate with regard to these nutrients of public health concern. Over- and under-reporting cannot be ruled out and may have introduced a certain bias to our results. An assessment of potential under-reporting with the doubly labeled water (DLW) method or 24-h urinary nitrogen method would have added to the quality of our work [49, 50]. However, 24-h urinary nitrogen data was only available for a very small subpopulation (approximately 750 individuals per NHANES cycles) [51], and DLW was not routinely performed in the NHANES.

Whether the estimated energy intake reflects the “*true*” habitual energy intake of the examined individuals (who frequently reported special diets) may also be subject to a controversial debate. This may apply in particular for our analysis, where females reported a significantly lower energy intake as opposed to males. The higher frequency of special diets in females, which often restrict daily energy intake, could play a pivotal role and serve as a potential explanation.

It is well known that dietary data from large surveys are often subject to bias. Some researchers go even as far as arguing that NHANES data are physiologically implausible and inadmissible as scientific evidence [52]. While we do not share this point of view, we clearly acknowledge that the data’s precision may be compromised by potential bias frequently observed in people with an unhealthy body weight. Against the background of the need for additional high-quality nutrition studies dedicated to collecting comprehensive dietary data from individuals with MO [13], new and innovative approaches are warranted. A rigid pre-selection based on energy intake criteria may not be suitable in such a case. This may be particularly true when it comes to special diets.

Special diets remain prevalent in the United States, with 17.1% of U.S. adults aged 20 and over reporting a special diet between 2015 and 2018 [53]. In our selective sample of NHANES participants with MO, this percentage was even higher and accounted for almost 28% of the examined population. Not considering those individuals (in order to exclude individuals with low and potentially unreliable or untypical energy intake from the analysis) would have introduced additional biases (e.g., selection bias). As such, we included this important population group. Nutrient intake data analyses in said individuals revealed significant differences when compared to those not on a special diet. This applied particularly for the total energy intake, carbohydrate intake and sodium intake. Regrettably, no data was available with regard to diet duration. A reservation must be made, however, that sub-analyses by special dietary pattern were deemed infeasible due to the low prevalence of some dietary patterns (e.g., the low-fat diet).

The present descriptive epidemiological study might be of high relevance from a public health nutrition perspective. It reveals (and reiterates) important sociodemographic aspects related to MO. One particular example includes socioeconomic disparities and nutrient intakes, given that the results indicated that lower incomes were more prevalent in higher BMI groups. It is well known that lower food expenditure mediates less-healthy choices in individuals with a lower socioeconomic status (SES) [54], and a recent study suggest that individuals living in low SES neighbourhoods with at least two fast-food outlets within 1 km of their residential address had a more unfavorable body weight than their peers with no fast-food outlets within 1 km distance [55]. Moreover, our analysis shows that the alignment with national dietary guidelines in individuals with MO appears to be low, particularly with regard to nutrients of public health concern. Finally, our results revealed that special diets remain prevalent in this part of the population, with almost every third person reporting some kind of special diet. Whether these diets actually contributed to MO or whether they were helpful in inducing weight loss was not ascertainable from our data.

The underlying dataset from the NHANES including its special (complex, multistage, clustered) sampling design and the comparison to the DGA are major strengths of this analysis. Unlike in many other studies, the data source is not restricted to a single institution. To the contrary, NHANES allows for nationally representative assessments and is thus of particular high value from a public health nutrition perspective. The fact that nutrient intake data was reported in a stratified manner is an additional asset. Nevertheless, our approach has weaknesses which we transparently acknowledge. Including those participants on a special diet and potential under-reporters (e.g., men with an energy intake below 800 kcal/d) may be problematic. Then again, the data was considered reliable as per the NHANES dietary module, and it is not inconceivable that energy intake is heavily restricted on some weight-loss diets. Further to that, the presented data reflects the actually reported *status quo*. In addition, we have not included physical activity data for our sample. While this aspect would have enriched the analysis, we refrained from doing so to avoid excluding additional NHANES participants with an incomplete dataset (and thus reducing the overall sample size). The number of participants with a history of weight loss surgery (as assessed by the question WHD080U) was small in the examined NHANES cycles and included approximately 20 participants per cycle [56]. A larger bias was thus deemed inconceivable. Sarcopenic obesity (characterized by the coexistence of loss of skeletal muscle mass and function and an excess of adipose tissue [57]) was not specifically examined in this descriptive nutritional epidemiology analysis, and should be subject to future investigations. Finally, our approach was descriptive in nature and did not include health-related outcomes. Thus, our results do not allow for statements as to *why* the investigated individuals were obese. To the contrary, we gathered data to describe sociodemographic and nutritional patterns in individuals with MO that may be used for targeted public health nutrition interventions, such as large-scale informational campaigns, “food-is-medicine” interventions, tailored meal programs, and targeted population-level food policies and programs.

## Conclusions

Females are more frequently affected by MO as compared to males. Regardless of sex, however, a low alignment with national dietary guidelines was observed. This applied in particular to the nutrients of public health concern, for which adequate intakes were not met by most NHANES participants with MO. Special diets remain prevalent among morbidly obese NHANES participants, with almost 30% reporting at least one special diet.

## Supplementary Information

**Supplementary Material 1. **

**Supplementary Material 2. **

**Supplementary Material 3. **

## Data Availability

Data is publicly available online (https://wwwn.cdc.gov/nchs/nhanes/Default.aspx). The datasets used and analyzed during the current study are available from the corresponding author on reasonable request.

## Abbreviations

AA: Associate of Arts
BMI: Body Mass Index
DHHS: Department of Health and Human Services
DLA: Doubly Labeled Water
DNG: Daily Nutritional Goals
DGA: Dietary Guidelines for Americans
GED: General Educational Development diploma
MO: Morbid Obesity
NCHS: National Center for Health Statistics
NHANES: National Health and Nutrition Examination Surveys
SES: Socioeconomic Status
USDA: US Department of Agriculture
