# Supplementary figures and images for: Special Diets and Nutrient Intakes in Morbidly Obese US Adults in Comparison to the 2020–2025 Dietary Guidelines for Americans
Source: Nutr J. 2025 Mar 8;24:37. doi: 10.1186/s12937-025-01088-7 (PMC11889755; doi:10.1186/s12937-025-01088-7)

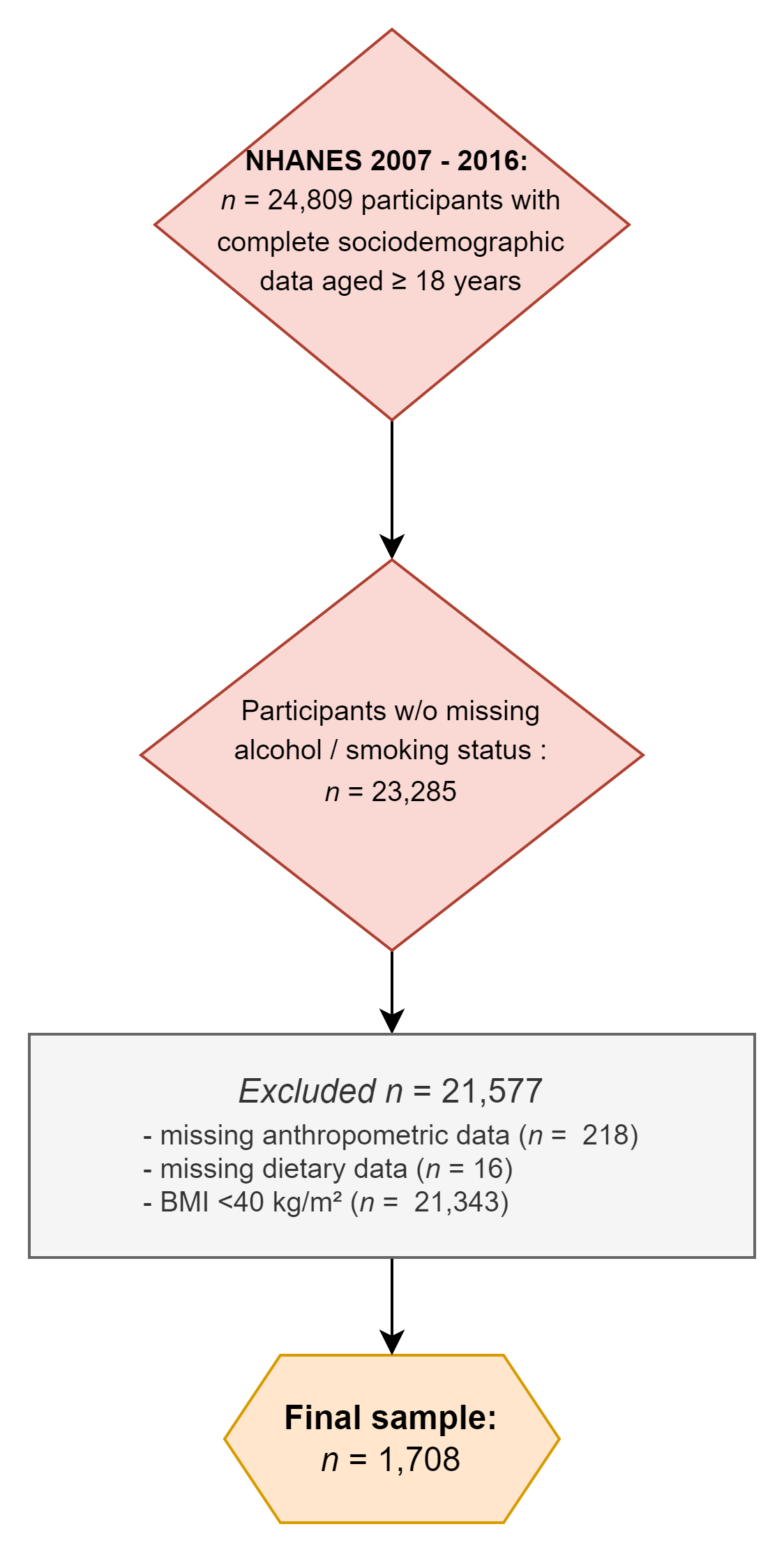

Supplement: Supplementary file 1 — Supplementary Material 1. [file 12937_2025_1088_MOESM1_ESM.png]

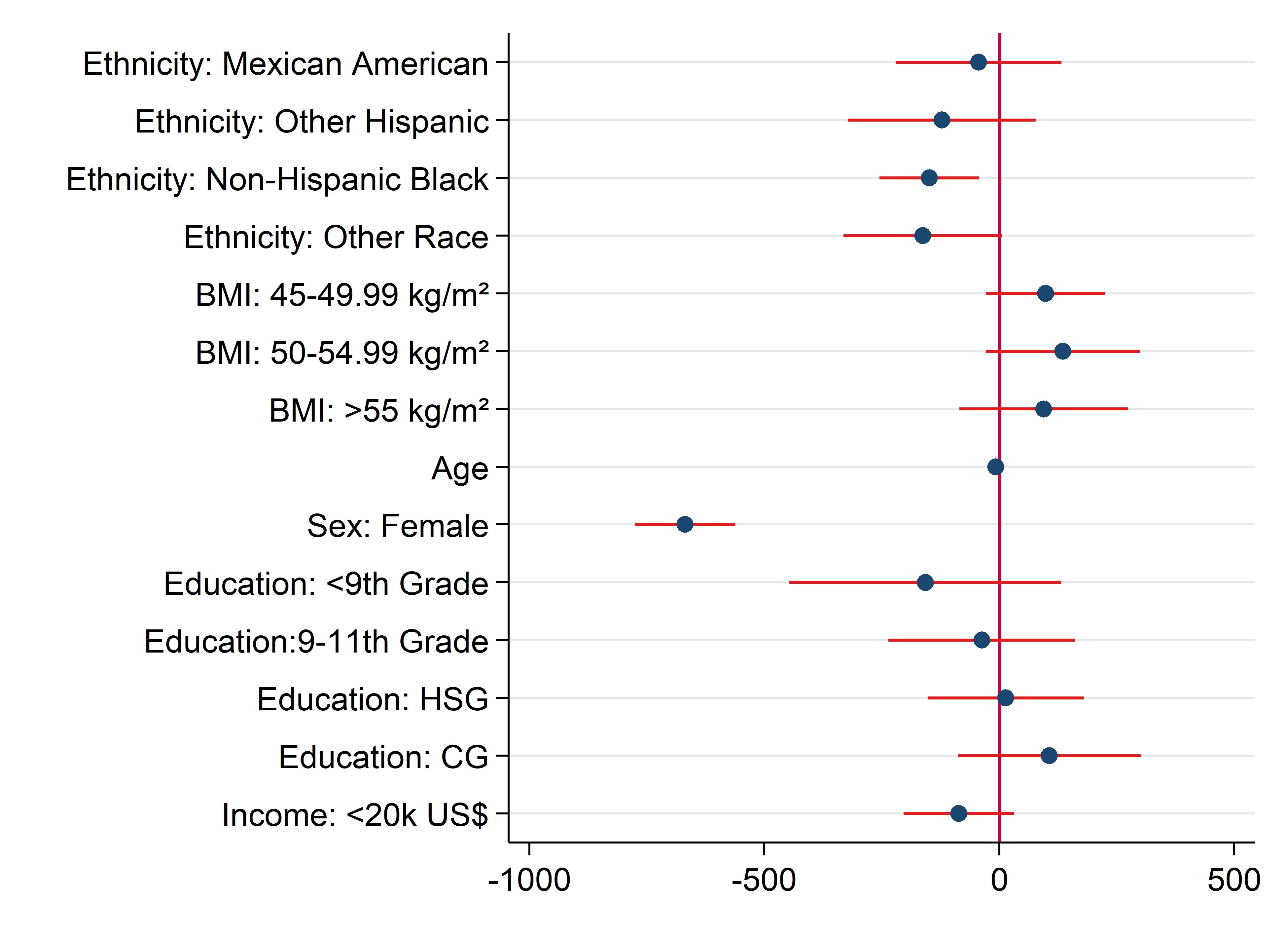

Supplement: Supplementary file 2 — Supplementary Material 2. [file 12937_2025_1088_MOESM2_ESM.jpg]
