# Supplementary material for: Special Diets and Nutrient Intakes in Morbidly Obese US Adults in Comparison to the 2020–2025 Dietary Guidelines for Americans
Source: Nutr J. 2025 Mar 8;24:37. doi: 10.1186/s12937-025-01088-7 (PMC11889755; doi:10.1186/s12937-025-01088-7)
Supplement: Supplementary file 3 — Supplementary Material 3. [file 12937_2025_1088_MOESM3_ESM.docx]

# Supplementary Tables

## Supplementary Table 1

Supplementary Table 1: Reporting of special diets in the unweighted cohort of *n* = 1,708 NHANES participants with morbid obesity

| Special diet | Number of mentions |
| --- | --- |
| Total | *n* = 456 |
| Weight loss or low calorie diets | *n* = 288 |
| Low fat/Low cholesterol diet | *n* = 46 |
| Low salt/Low sodium diet | *n* = 52 |
| Sugar free/Low sugar diet | *n* = 14 |
| Low carbohydrate diet | *n* = 33 |
| High fiber diet | *n* = 1 |
| Diabetic diet | *n* = 94 |
| High protein diet | *n* = 12 |
| Other special diet | *n* = 10 |

Supplementary Table 1 legend: According to the NHANES, more than one self-reported special diet per participant was possible, thus the number of individuals on a special diet does not correspond with the number of the individually reported special diets.

## Supplementary Table 2

Supplementary Table 2: Nutrient intakes in the entire cohort of *n* = 1,708 participants

| Nutrient | **Whole Sample** *(n =1,708)* | |
| --- | --- | --- |
|  | mean | CI |
| Energy intake (kcal/d) | 2113.16 | 2057.26-2169.07 |
| Protein (%kcal) | 16.04 | 15.66-16.42 |
| Protein (g) | 81.99 | 79.49-84.49 |
| Carbohydrate (%kcal) | 48.08 | 47.27-48.89 |
| Carbohydrate (g) | 251.57 | 244.12-259.02 |
| Fiber (g) | 15.86 | 15.08-16.64 |
| Total lipid (%kcal) | 35.27 | 34.51-36.04 |
| Saturated Fatty Acids (%kcal) | 11.57 | 11.22-11.92 |
| 18:2 Linoleic acid (g) | 16.92 | 16.14-17.70 |
| 18:3 Linolenic acid (g) | 1.73 | 1.65-1.82 |
| Calcium (mg) | 933.96 | 897.28-970.65 |
| Iron (mg) | 14.29 | 13.76-14.82 |
| Magnesium (mg) | 278.37 | 268.12-288.63 |
| Phosphorus (mg) | 1345.54 | 1303.23-1387.86 |
| Potassium (mg) | 2529.55 | 2426.16-2632.94 |
| Sodium (mg) | 3613.88 | 3498.64-3729.13 |
| Zinc (mg) | 11.02 | 10.60-11.45 |
| Vitamin A (RAE mcg) | 583.10 | 550.98-615.23 |
| Vitamin E (mg ATd) | 7.97 | 7.56-8.39 |
| Vitamin D (IUDd) | 161.90 | 147.19-176.62 |
| Vitamin C (mg) | 72.90 | 67.19-78.60 |
| Thiamin (mg) | 1.54 | 1.49-1.59 |
| Riboflavin (mg) | 2.05 | 1.97-2.12 |
| Niacin (mg) | 24.87 | 24.06-25.68 |
| Vitamin B-6 (mg) | 1.92 | 1.83-2.01 |
| Vitamin B-12 (mcg) | 4.91 | 4.57-5.25 |
| Choline (mg) | 323.48 | 312.04-334.91 |
| Vitamin K (mcg) | 107.15 | 96.93-117.37 |
| Folate (mcg DFEd) | 499.56 | 476.25-522.87 |

## Supplementary Table 3

Supplementary Table 3: Nutrient intakes in the entire cohort of *n* = 1,708 participants by sex

| Nutrient | **Males** *(n =575)* | | **Females** *(n =1,133)* | |
| --- | --- | --- | --- | --- |
|  | mean | CI | mean | CI |
| Energy intake (kcal/d) | 2560.99 | 2459.19-2662.79 | 1878.33 | 1829.33-1927.33 |
| Protein (%kcal) | 16.19 | 15.48-16.89 | 15.96 | 15.56-16.36 |
| Protein (g) | 100.52 | 95.88-105.16 | 72.28 | 70.14-74.42 |
| Carbohydrate (%kcal) | 46.57 | 45.06-48.08 | 48.87 | 47.82-49.93 |
| Carbohydrate (g) | 296.51 | 280.39-312.62 | 228.00 | 220.75-235.25 |
| Fiber (g) | 18.96 | 17.35-20.57 | 14.24 | 13.60-14.87 |
| Total lipid (%kcal) | 35.66 | 34.46-36.87 | 35.07 | 34.21-35.93 |
| Saturated Fatty Acids (%kcal) | 11.67 | 11.20-12.15 | 11.51 | 11.10-11.93 |
| 18:2 Linoleic acid (g) | 20.41 | 18.73-22.09 | 15.09 | 14.49-15.70 |
| 18:3 Linolenic acid (g) | 2.06 | 1.87-2.24 | 1.56 | 1.49-1.63 |
| Calcium (mg) | 1097.99 | 1028.63-1167.35 | 847.95 | 814.96-880.94 |
| Iron (mg) | 17.40 | 16.32-18.47 | 12.66 | 12.24-13.08 |
| Magnesium (mg) | 334.09 | 316.25-351.94 | 249.16 | 240.79-257.52 |
| Phosphorus (mg) | 1624.24 | 1550.96-1697.52 | 1199.40 | 1165.66-1233.14 |
| Potassium (mg) | 3082.69 | 2893.63-3271.75 | 2239.49 | 2162.16-2316.82 |
| Sodium (mg) | 4384.78 | 4128.33-4641.24 | 3209.63 | 3119.27-3299.99 |
| Zinc (mg) | 13.74 | 12.96-14.52 | 9.59 | 9.23-9.96 |
| Vitamin A (RAE mcg) | 618.27 | 570.86-665.68 | 564.66 | 521.31-608.02 |
| Vitamin E (mg ATd) | 9.40 | 8.63-10.17 | 7.22 | 6.81-7.64 |
| Vitamin D (IUDd) | 192.80 | 163.74-221.85 | 145.70 | 131.11-160.29 |
| Vitamin C (mg) | 81.92 | 68.96-94.87 | 68.16 | 62.85-73.48 |
| Thiamin (mg) | 1.86 | 1.76-1.97 | 1.36 | 1.32-1.41 |
| Riboflavin (mg) | 2.55 | 2.39-2.71 | 1.79 | 1.72-1.85 |
| Niacin (mg) | 31.25 | 29.56-32.94 | 21.52 | 20.77-22.28 |
| Vitamin B-6 (mg) | 2.44 | 2.26-2.62 | 1.65 | 1.57-1.73 |
| Vitamin B-12 (mcg) | 6.00 | 5.44-6.55 | 4.34 | 3.99-4.69 |
| Choline (mg) | 396.32 | 375.44-417.20 | 285.28 | 275.11-295.45 |
| Vitamin K (mcg) | 113.63 | 95.32-131.95 | 103.76 | 91.43-116.08 |
| Folate (mcg DFEd) | 601.89 | 556.08-647.70 | 445.89 | 423.91-467.88 |

## Supplementary Table 4

Supplementary Table 4: Nutrient intakes by self-reported special diet: whole sample

| Nutrient | **Special Diet: Yes**  *(n = 456)* | | **Special Diet: No**  *(n = 1252)* | | ***p*-value** |
| --- | --- | --- | --- | --- | --- |
|  | mean | CI | mean | CI |  |
| Energy intake (kcal/d) | 1883.75 | 1787.18-1980.32 | 2200.95 | 2133.10-2267.89 | **<0.001** |
| Protein (%kcal) | 17.38 | 16.57-18.20 | 15.52 | 15.21-15.83 | **<0.001** |
| Protein (g) | 78.23 | 72.98-83.48 | 83.43 | 80.83-86.04 | 0.069 |
| Carbohydrate (%kcal) | 46.91 | 45.22-48.60 | 48.53 | 47.73-49.33 | 0.067 |
| Carbohydrate (g) | 217.95 | 205.33 | 264.43 | 255.25-273.62 | **<0.001** |
| Fiber (g) | 16.06 | 14.29-17.82 | 15.79 | 15.01-16.52 | 0.773 |
| Total lipid (%kcal) | 35.50 | 34.07-36.92 | 35.19 | 34.39-35.99 | 0.675 |
| Saturated Fatty Acids (%kcal) | 11.35 | 10.89-11.80 | 11.66 | 11.23-12.09 | 0.308 |
| 18:2 Linoleic acid (g) | 15.42 | 14.04-16.79 | 17.50 | 16.60-18.40 | **0.010** |
| 18:3 Linolenic acid (g) | 1.62 | 1.46-1.78 | 1.77 | 1.69-1.86 | 0.056 |
| Calcium (mg) | 896.25 | 844.49-948.02 | 948.39 | 903.38-993.41 | 0.117 |
| Iron (mg) | 13.53 | 12.48-14.58 | 14.58 | 13.99-15.17 | 0.249 |
| Magnesium (mg) | 275.32 | 258.07-292.57 | 279.54 | 267.68-291.41 | 0.673 |
| Phosphorus (mg) | 1266.47 | 1193.57-1339.37 | 1375.80 | 1328.75-1422.85 | **0.008** |
| Potassium (mg) | 2469.37 | 2309.94-2628.80 | 2552.58 | 2434.24-2670.91 | 0.361 |
| Sodium (mg) | 3310.09 | 3123.15-3497.03 | 3730.13 | 3592.38-3867.87 | **<0.001** |
| Zinc (mg) | 10.32 | 9.60-11.04 | 11.29 | 10.76-11.82 | **0.039** |
| Vitamin A (RAE mcg) | 609.92 | 558.07-661.77 | 572.84 | 529.17-616.52 | 0.322 |
| Vitamin E (mg ATd) | 8.25 | 7.39-9.10 | 7.87 | 7.45-8.28 | 0.396 |
| Vitamin D (IUDd) | 165.03 | 139.02-191.05 | 160.70 | 143.43-177.98 | 0.779 |
| Vitamin C (mg) | 74.92 | 65.35-84.50 | 72.12 | 64.70-79.55 | 0.665 |
| Thiamin (mg) | 1.44 | 1.34-1.54 | 1.58 | 1.52-1.63 | **0.012** |
| Riboflavin (mg) | 1.97 | 1.85-2.09 | 2.08 | 1.99-2.17 | 0.103 |
| Niacin (mg) | 23.25 | 21.87-24.63 | 25.49 | 24.55-26.43 | **0.007** |
| Vitamin B-6 (mg) | 1.87 | 1.72-2.02 | 1.94 | 1.84-2.04 | 0.406 |
| Vitamin B-12 (mcg) | 4.44 | 4.00-4.88 | 5.09 | 4.67-5.51 | **0.029** |
| Choline (mg) | 315.85 | 289.11-342.60 | 326.39 | 314.92-337.87 | 0.463 |
| Vitamin K (mcg) | 123.63 | 101.61-145.64 | 100.85 | 89.81-111.90 | 0.067 |
| Folate (mcg DFEd) | 469.25 | 419.67-518.83 | 511.15 | 486.08-536.22 | 0.128 |

## Supplementary Table 5

Supplementary Table 5: Nutrient intakes by special diet (SD): males only

| Nutrient | **Special Diet: Yes**  *(n = 144)* | | **Special Diet: No**  *(n = 431)* | | ***p*-value** |
| --- | --- | --- | --- | --- | --- |
|  | mean | CI | mean | CI |  |
| Energy intake (kcal/d) | 2301.53 | 2094.36-2508.71 | 2635.74 | 2515.53-2755.95 | **0.009** |
| Protein (%kcal) | 17.02 | 15.17-18.88 | 15.94 | 15.31-16.58 | 0.251 |
| Protein (g) | 94.83 | 81.17-108.48 | 102.15 | 98.05-106.26 | 0.293 |
| Carbohydrate (%kcal) | 45.92 | 42.64-49.21 | 46.76 | 45.32-48.19 | 0.603 |
| Carbohydrate (g) | 254.90 | 230.14-279.66 | 308.50 | 289.52-327.47 | **<0.001** |
| Fiber (g) | 20.28 | 15.79-24.78 | 18.58 | 17.11-20.05 | 0.462 |
| Total lipid (%kcal) | 35.91 | 33.23-38.60 | 35.59 | 34.48-36.70 | 0.800 |
| Saturated Fatty Acids (%kcal) | 11.23 | 10.44-12.03 | 11.80 | 11.28-12.32 | 0.180 |
| 18:2 Linoleic acid (g) | 19.15 | 16.11-22.18 | 20.77 | 18.90-22.64 | 0.334 |
| 18:3 Linolenic acid (g) | 2.00 | 1.62-2.37 | 2.08 | 1.90-2.25 | 0.653 |
| Calcium (mg) | 984.77 | 873.84-1095.70 | 1130.61 | 1046.83-1214.40 | **0.039** |
| Iron (mg) | 16.15 | 13.75-18.55 | 17.75 | 16.52-18.94 | 0.080 |
| Magnesium (mg) | 326.65 | 292.61-360.68 | 336.24 | 316.10-356.38 | 0.618 |
| Phosphorus (mg) | 1511.76 | 1348.15-1675.37 | 1656.65 | 1579.35-1733.94 | 0.096 |
| Potassium (mg) | 2956.24 | 2636.37-3276.12 | 3119.12 | 2898.69-3339.55 | 0.390 |
| Sodium (mg) | 4040.30 | 3568.28-4512.32 | 4484.03 | 4213.13-4754.94 | 0.069 |
| Zinc (mg) | 12.81 | 11.21-14.40 | 14.01 | 13.13-14.89 | 0.187 |
| Vitamin A (RAE mcg) | 684.39 | 569.43-799.34 | 599.22 | 547.79-650.64 | 0.189 |
| Vitamin E (mg ATd) | 10.43 | 8.87-12.00 | 9.11 | 8.27-9.94 | 0.128 |
| Vitamin D (IUDd) | 177.86 | 147.37-208.36 | 197.10 | 160.56-233.64 | 0.429 |
| Vitamin C (mg) | 85.92 | 66.53-105.31 | 80.77 | 65.61-95.94 | 0.663 |
| Thiamin (mg) | 1.68 | 1.43-1.93 | 1.92 | 1.81-2.03 | 0.073 |
| Riboflavin (mg) | 2.33 | 2.12-2.55 | 2.61 | 2.42-2.81 | 0.057 |
| Niacin (mg) | 27.86 | 24.32-31.39 | 32.23 | 30.37-34.08 | **0.027** |
| Vitamin B-6 (mg) | 2.28 | 1.94-2.61 | 2.49 | 2.28-2.69 | 0.271 |
| Vitamin B-12 (mcg) | 5.15 | 4.46-5.85 | 6.24 | 5.59-6.89 | **0.015** |
| Choline (mg) | 414.23 | 341.62-486.83 | 391.17 | 373.61-408.72 | 0.545 |
| Vitamin K (mcg) | 124.72 | 97.76-151.69 | 110.44 | 88.60-132.28 | 0.407 |
| Folate (mcg DFEd) | 581.93 | 468.79-695.07 | 607.64 | 559.23-656.05 | 0.675 |

## Supplementary Table 6

Supplementary Table 6: Nutrient intakes by special diet (SD): females only

| Nutrient | **Special Diet: Yes**  *(n = 312)* | | **Special Diet: No**  *(n = 821)* | | ***p*-value** |
| --- | --- | --- | --- | --- | --- |
|  | mean | CI | mean | CI |  |
| Energy intake (kcal/d) | 1722.86 | 1634.31-1811.41 | 1946.42 | 1885.55-2007.29 | **<0.001** |
| Protein (%kcal) | 17.52 | 16.60-18.44 | 15.28 | 14.91-15.64 | **<0.001** |
| Protein (g) | 71.84 | 67.14-76.53 | 72.47 | 70.02-74.93 | 0.818 |
| Carbohydrate (%kcal) | 47.29 | 45.44-49.15 | 49.56 | 48.43-50.70 | **0.027** |
| Carbohydrate (g) | 203.72 | 190.98-216.46 | 238.64 | 229.58-247.69 | **<0.001** |
| Fiber (g) | 14.43 | 13.03-15.83 | 14.15 | 13.49-14.81 | 0.716 |
| Total lipid (%kcal) | 35.34 | 33.97-36.70 | 34.95 | 33.96-35.94 | 0.620 |
| Saturated Fatty Acids (%kcal) | 11.39 | 10.87-11.90 | 11.57 | 11.02-12.12 | 0.627 |
| 18:2 Linoleic acid (g) | 13.98 | 12.73-15.23 | 15.58 | 14.88-16.29 | **0.033** |
| 18:3 Linolenic acid (g) | 1.47 | 1.32-1.63 | 1.60 | 1.53-1.67 | 0.127 |
| Calcium (mg) | 862.17 | 803.89-920.44 | 841.72 | 799.02-884.42 | 0.592 |
| Iron (mg) | 12.52 | 11.69-13.35 | 12.72 | 12.22-13.22 | 0.687 |
| Magnesium (mg) | 255.55 | 238.33-272.77 | 246.35 | 237.59-255.12 | 0.328 |
| Phosphorus (mg) | 1172.01 | 1101.44-1242.58 | 1211.39 | 1172.02-1250.76 | 0.349 |
| Potassium (mg) | 2281.87 | 2116.89-2446.85 | 2220.93 | 2144.32-2297.53 | 0.485 |
| Sodium (mg) | 3028.89 | 2851.58-3206.20 | 3288.79 | 3170.47-3407.12 | **0.030** |
| Zinc (mg) | 9.36 | 8.69-10.04 | 9.70 | 9.24-10.16 | 0.442 |
| Vitamin A (RAE mcg) | 581.25 | 515.07-647.42 | 557.40 | 500.90-613.91 | 0.596 |
| Vitamin E (mg ATd) | 7.40 | 6.42-8.39 | 7.14 | 6.75-7.53 | 0.616 |
| Vitamin D (IUDd) | 160.09 | 127.73-192.45 | 139.40 | 123.98-154.82 | 0.256 |
| Vitamin C (mg) | 70.69 | 60.52-80.85 | 67.06 | 59.59-74.53 | 0.609 |
| Thiamin (mg) | 1.34 | 1.25-1.43 | 1.37 | 1.32-1.43 | 0.561 |
| Riboflavin (mg) | 1.83 | 1.69-1.97 | 1.77 | 1.69-1.84 | 0.452 |
| Niacin (mg) | 21.48 | 20.16-22.80 | 21.54 | 20.59-22.49 | 0.942 |
| Vitamin B-6 (mg) | 1.71 | 1.56-1.87 | 1.62 | 1.52-1.72 | 0.332 |
| Vitamin B-12 (mcg) | 4.17 | 3.67-4.67 | 4.41 | 3.94-4.89 | 0.505 |
| Choline (mg) | 277.97 | 257.71-298.24 | 288.48 | 275.71-301.25 | 0.410 |
| Vitamin K (mcg) | 123.20 | 95.66-150.75 | 95.24 | 84.62-105.86 | 0.046 |
| Folate (mcg DFEd) | 425.86 | 387.05-464.67 | 454.67 | 427.74-481.60 | 0.235 |

## Supplementary Table 7

Supplementary Table 7: Multivariate linear regression model to estimate energy intakes in morbidly obese NHANES participants

| Independent Variable | beta-coefficient | 95%-CI | p-value |
| --- | --- | --- | --- |
|  |  |  |  |
| Ethnicity: Mexican America | -44.22 | -220.47 - 132.03 | 0.619 |
| Ethnicity: Other Hispanic | -122.10 | -322.49 - 78.28 | 0.229 |
| Ethnicity: Non-Hispanic Black | -149.07 | -254.68 – (-43.45) | ***0.006*** |
| Ethnicity: Other Race | -163.17 | -331.82 - 5.49 | 0.058 |
|  |  |  |  |
| BMI: 45-49.99 | 98.71 | -27.86 - 225.29 | 0.125 |
| BMI: 50-54.99 | 135.14 | -28.48 - 298.74 | 0.104 |
| BMI: >55 | 94.62 | -84.95 - 274.20 | 0.297 |
|  |  |  |  |
| Age in years | -7.57 | -11.75 - (-3.40) | ***0.001*** |
|  |  |  |  |
| Sex: Female | -668.91 | -774.76 - (-563.07) | ***<0.001*** |
|  |  |  |  |
| Education: <9th Grade | -157.50 | -446.69 - 131.70 | 0.282 |
| Education: 9-11th Grade | -37.27 | -235.74 - 161.19 | 0.710 |
| Education: High school graduate/GED d | 13.95 | -152.22 - 180.11 | 0.868 |
| Education: College graduate or above | 106.62 | -87.40 - 300.65 | 0.277 |
|  |  |  |  |
| Income: <20000 US$ | -86.20 | -203.70 - 31.29 | 0.148 |

Supplementary Table 7: Supplementary Table 7 displays a multivariate linear regression model to estimate energy intakes in morbidly obese NHANES participants. The reference categories were as follows: Ethnicity: Non-Hispanic White; BMI 40-44.99; Sex: Male; Education: Some college or AA degree; Income: >20000 US$. Post regression, we used Stata’s marginsplot function to visualize statistics from this model and to display marginal predicted values (see Figure 1).
